# Conventional Biomarkers for Predicting Clinical Outcomes in Patients with Heart Disease

**DOI:** 10.3390/life12122112

**Published:** 2022-12-15

**Authors:** Ion-Bogdan Mănescu, Krisztina Pál, Silvia Lupu, Minodora Dobreanu

**Affiliations:** 1 Clinical Laboratory, County Emergency Clinical Hospital of Targu Mures, 540136 Targu Mures, Romania; 2 Department of Laboratory Medicine, George Emil Palade University of Medicine, Pharmacy, Science, and Technology of Targu Mures, 540142 Targu Mures, Romania; 3 Internal Medicine V, George Emil Palade University of Medicine, Pharmacy, Science, and Technology of Targu Mures, 540142 Targu Mures, Romania; 4 1st Department of Cardiology, Emergency Institute for Cardiovascular Disease and Heart Transplant of Targu Mures, 540136 Targu Mures, Romania; 5 Center for Advanced Medical and Pharmaceutical Research, George Emil Palade University of Medicine, Pharmacy, Science, and Technology of Targu Mures, 540139 Targu Mures, Romania

**Keywords:** coronary artery disease, acute coronary syndrome, heart failure, biomarkers

## Abstract

Atherosclerosis is the main cause of cardiovascular disease worldwide. The progression of coronary atherosclerosis leads to coronary artery disease, with impaired blood flow to the myocardium and subsequent development of myocardial ischemia. Acute coronary syndromes and post-myocardial infarction heart failure are two of the most common complications of coronary artery disease and are associated with worse outcomes. In order to improve the management of patients with coronary artery disease and avoid major cardiovascular events, several risk assessment tools have been developed. Blood and imaging biomarkers, as well as clinical risk scores, are now available and validated for clinical practice, but research continues. The purpose of the current paper is to provide a review of recent findings regarding the use of humoral biomarkers for risk assessment in patients with heart disease.

## 1. Introduction

Atherosclerosis is the main cause of cardiovascular disease (CVD) worldwide. The progression of coronary atherosclerosis leads to coronary artery disease (CAD), with impaired blood flow to the myocardium and subsequent development of myocardial ischemia. Acute coronary syndrome (ACS) and post-myocardial infarction (MI) heart failure (HF) are two of the most common complications of CAD. Considerable progress has been made regarding risk assessment in patients presenting with ACS, and several blood and imaging biomarkers, as well as clinical risk scores, are now available and validated for clinical practice. However, the quest for ever improved biomarkers in this field continues, driven by increased life expectancy, growing patient heterogeneity, accumulation of clinical data, and booming biotechnological progress.

In this article, we aimed to review recent and relevant literature regarding the value of conventional humoral biomarkers as predictors of outcome in patients with CAD. The pathophysiological mechanisms behind each biomarker’s predictive role are beyond the purpose of this paper and are only briefly approached.

## 2. Materials and Methods

A systematic electronic literature search was conducted using the PubMed database on 7th September 2022 to identify recent and relevant papers regarding humoral biomarkers with predictive value for clinical outcomes in patients with CVD, mainly myocardial ischemia-related conditions and ischemia-related heart failure.

The following formula was used for searching the database: “(myocardial ischemia) AND (heart failure) AND (prognosis OR outcome OR prognostic factor OR prediction) AND (biomarker OR marker)”; and a total of 3206 results were retrieved. After filtering results based on year of publication (2015–2022), article type, and species (humans), a total of 428 original articles were found. Secondary research articles (reviews, systematic reviews, or meta-analyses) were excluded.

The 428 articles were analyzed independently by two medical doctors who further excluded: non-human studies, non-clinical studies, articles focusing solely on non-humoral biomarkers, and articles where the predictive role of the biomarker was not studied. Articles included in the final analysis were divided into two categories based on the investigated biomarker type—conventional or emerging. The present review includes 44 of the selected articles which address mainly conventional biomarkers. The remaining articles will be included in a second part of this work, focusing on emerging biomarkers. Articles investigating multiple biomarkers have been cited in all relevant sections.

All biomarkers reviewed in this article are approached with the same methodology and share equal emphasis. However, it should be stated that some of these biomarkers are more relevant than others in current clinical practice. The number of references for each biomarker should not necessarily be considered an indicator of its relative importance.

Relevant statistical data are inserted in separate tables, in order to facilitate reading.

## 3. Results

### 3.1. Natriuretic Peptides (NPs)

NPs are hormones produced by cardiomyocytes in response to stretching of the myocardium. Although several NPs have been identified, only B-type NPs are currently relevant in routine clinical practice. Thus, brain natriuretic peptide (BNP) and the biologically inactive N-terminal fragment in the prohormone (NT-proBNP) are widely used in the diagnostic process of HF. Low NP levels are especially useful to rule out HF [1], but increased levels may occur in other cardiovascular and non-cardiovascular conditions. Moreover, NPs have proved reliable markers of major adverse cardiovascular events (MACE) in patients with CVD. 

#### 3.1.1. Predictors of NPs Levels

Several predictors for NPs levels have been identified. Data from 885 patients with suspected stable angina who were included in the Scottish Computed Tomography of the Heart trial showed that age and atherosclerotic burden were independent predictors of both increased BNP and troponin, while female sex and left ventricular volume were independent predictors of increased BNP, but not troponin [2]. Another study identified age, atrial fibrillation, body mass index, renal dysfunction, and left atrium size as predictors of NT-proBNP levels in 2039 patients with HF [3].

#### 3.1.2. NPs in the Absence of CVD

Long-term cardiovascular outcomes were shown to be predicted by baseline NT-proBNP levels even in the absence of CVD. In a substudy of the Multi-Ethnic Study of Atherosclerosis (MESA) trial, including 6814 participants without CVD followed for 12 years, among 735 different variables, NT-proBNP had the 4th, 3rd, and 1st relative variable importance as predictor of CAD, all CVD types, and HF, respectively [4]. In another MESA substudy with a similar number of participants (n = 6781), elevated NT-proBNP was a significant predictor of incident HF with preserved ejection fraction (HFpEF) [5].

#### 3.1.3. NPs Prior to ACS

Premorbid levels of NT-proBNP, measured 5.8 years prior to incident MI, were associated with adverse outcomes in a substudy of 1054 participants from the Atherosclerosis Risk in Communities Study (ARIC) [6]. Patients with higher levels of NT-proBNP had an increased risk for composite outcome (all-cause mortality, cardiovascular mortality, recurrent MI, HF, stroke) both within 30 days and >30 days after incident MI [6]. The same three NT-proBNP categories were also associated with individual outcomes, but only for all-cause mortality, cardiovascular mortality, and HF. These associations were more evident for NT-proBNP than hs-cTnT [6].

#### 3.1.4. NPs in ACS

Several studies have shown that NPs are valuable markers when measured at the time of or shortly after incident ACS. In a study on 505 patients suspected of ACS presenting within 4 h of chest pain onset, the levels of BNP signal peptide (BNPsp) measured within 24 h from admission did not discriminate between MI and unstable angina and failed to add to troponin [7]. However, BNPsp did identify patients with unstable angina among non-MI patients, and helped predict outcomes when added to a composite parameter (the UARatio). The UARatio included BNPsp, NT-proBNP, potassium and white blood cell count, and predicted stroke and HF within one year, in patients with unstable angina and normal ECG [7].

In a small study on 270 patients, higher midregional proatrial natriuretic peptide (MR-proANP) levels were recorded on admission in type 2 MI, by comparison with type 1 MI [8]. MR-proANP performed better than cTnI (cardiac troponin I) at differentiating type 2 MI. Moreover, unlike cTnI, MR-proANP was predictive for 180-day outcomes (mortality and MACEs, a composite of acute myocardial infarction, unstable angina pectoris, reinfarction, heart failure, and stroke) regardless of the type of MI. 

A secondary analysis of the PLATelet inhibition and patient Outcomes (PLATO) biomarker study on 17,905 patients presenting with ACS (37.6% with ST-segment elevation myocardial infarction) showed that NT-proBNP levels measured at a median of 10 h after admission was the strongest marker of all-cause mortality, death due to MI, and sudden cardiac death/arrhythmia [9].

In addition to that, a retrospective study on 426 patients admitted with first incident acute MI treated with primary percutaneous coronary angioplasty (PCI) [10] reported that NT-proBNP levels measured within 24 h from admission predicted HFpEF during hospitalization. Moreover, NT-proBNP had higher predictive value by comparison with high-sensitivity C-reactive protein (hs-CRP), and the number of diseased vessels. Moreover, NPs levels measured at 24 h were better predictors of one-year mortality and/or hospitalization for acute HF than NPs at admission, in a study on 913 ST segment elevation MI (STEMI) patients treated by primary PCI [11]. NPs at 24 h also maintained their predictive value of the same outcomes after two and three years, respectively [11].

In a study on 431 STEMI patients, pre-PCI BNP levels were also shown to predict 1-year mortality, with reasonable sensitivity and specificity (cut-off ≥150.2 pg/mL, AUC 0.787 [0.687–0.888], 72.7% sensitivity, 78.5% specificity, *p* < 0.001), as well as other outcomes, such as hospitalization due to acute HF, combined mortality and/ or acute HF hospitalization, revascularization, and reinfarction [12]. Similar results were reported in a study on 64 patients with cardiogenic shock due to STEMI [13], in which NT-proBNP at 24 h from admission was an independent predictor of mortality, and in-hospital mortality (68% specificity, 76.5% sensitivity, 89% negative predictive value, and 46.4% positive predictive value, for a cut off value of 8582 pg/mL).

#### 3.1.5. NPs after ACS

According to existing data, the levels of NPs measured within days or months after an ACS may also be used to predict mortality and cardiovascular outcomes. A substudy of the Eplerenone Post-Acute Myocardial Infarction Heart Failure Efficacy and Survival Study (EPHESUS) provided evidence for the prognostic use of BNP in patients randomized at 3–14 days after acute MI. The study included 463 patients with reduced left ventricular ejection fraction (LVEF) ≤40%, and either congestive HF or diabetes mellitus [14]. In this study, higher baseline BNP was associated with the risk of all-cause death and the composite outcome of cardiovascular death or hospitalization for HF. Increased baseline BNP and one-month BNP change from baseline were also associated with an increased risk of hospitalization for HF, and cardiovascular death. Moreover, data form the Long-term Intervention with Pravastatin in Ischemic Heart Disease (LIPID) trial, collected from 7101 patients with a history of ACS 3–36 months before recruitment [15], showed that BNP >50.29 pg/mL doubled the risk of a future HF event over a five-year follow-up period. Similarly, a smaller study on 642 patients attending a cardiac rehabilitation program after ACS showed that (ln)NT-proBNP was among the most important predictors of all-cause and cardiac-related mortality in CAD patients after an ACS [16].

In the Examination of Cardiovascular Outcomes with Alogliptin versus Standard of Care (EXAMINE) trial, NT-proBNP levels were measured in 5154 type 2 diabetes mellitus patients at baseline (15–90 days post ACS) and again after six months [17]. In this trial, NT-proBNP was among the strongest predictors for HF outcomes, as a doubling of NT-proBNP levels was associated with HF hospitalization and cardiovascular death. Similar findings were provided by data from the Evaluation of Lixisenatide in Acute Coronary Syndrome (ELIXA) trial [18]. In 5525 patients with type 2 diabetes mellitus and recent ACS (up to 180 days), baseline BNP/ NT-proBNP levels were the best predictors for death, cardiovascular death, HF, and stroke, and second-best predictors for MI (after previous MI) [18]. The predictive strength of BNP alone was similar to that of base models without BNP in outcomes of death and cardiovascular death. Regarding the long-term prognostic value of NPs after ACS, a substudy of the Heart and Soul Study, involving 635 patients with stable CAD, showed that changes in plasma NT-proBNP over a five-year period were associated with higher risk of HF and cardiovascular death [19]. The Hellenic Heart Failure (HHF) study, which included 1000 patients with ACS, showed that BNP levels at the time of admission were independent predictors of mortality even after 10 years [20]. In stratified analysis, BNP levels predicted 10-year all-cause mortality in HFpEF and HF with midrange EF, but not in HF with reduced LVEF (HFrEF) [20]. Relevant statistical data for this section are presented in Table 1.

#### 3.1.6. NPs in HF

The value of NPs in patients with HF is well established, in a wide range of cardiovascular diseases, including CAD. We further present data from more recent trials.

In a retrospective cohort study on 2456 patients with at least one primary or secondary HF diagnosis during hospitalization or ER visit, NT-proBNP levels ≥411 pg/mL (median value) were not associated with higher risks for ischemic stroke and MI. However, patients with NT-proBNP levels above the threshold had higher risks of all-cause mortality [21]. Among patients with HFrEF, those with higher NT-proBNP levels (≥644 pg/mL for LVEF <40%; ≥534 pg/mL for LVEF 40–49%; ≥321 pg/mL for LVEF ≥50%) had a five-fold increased risk of ischemic stroke, while elevated NT-proBNP was associated with all-cause mortality in both HFpEF and HFrEF patients [21]. Another study on 2039 patients with HF reported that higher NT-proBNP was a risk predictor for combined HF readmission and all-cause death, independently of LVEF [3].

#### 3.1.7. NPs Improve Current Predictive Models

Although NPs have value as predictors of outcome when used alone, some studies show that they can also provide incremental value to already established predictive models.

For instance, MR-proANP helped better differentiate between type 2 and type 1 MI when added to a clinical model, together with three other biomarkers [8]. When added to the TIMI Risk Score for Secondary Prevention, premorbid NT-proBNP levels improved risk assessment for cardiovascular mortality, all-cause mortality, HF, recurrent MI, and stroke after MI [6]. NT-proBNP and D-dimers together provided incremental value when added to the GRACE score for all-cause death, while for MACEs (all-cause death, hospital admission for unstable angina, hospital admission for heart failure, nonfatal recurrent myocardial infarction, and stroke), the predictive value was improved when fibrinogen was also added to the model [22]. Similarly, the addition of BNP to the GRACE score in 913 STEMI patients treated by primary PCI predicted one-year mortality and/ or hospitalization for acute HF better than the original model [11]. The pre-PCI BNP levels increased predictive power for one-year mortality and combined one-year mortality and/ or hospitalization for acute HF when added to the TIMI score in STEMI patients [12]. Moreover, in a subanalysis of the LIPID trial, the addition of BNP to five other biomarkers included in a clinical risk model significantly improved the prediction of five-year HF occurrence in patients who experienced an ACS 3–36 months prior to randomization [15].

However, in patients with stable CAD, NT-proBNP did not improve the prognostic power of a clinical model based on traditional clinical risk factors, echocardiographic parameters, ischemia, other biomarkers, and New York Heart Association class [19].

With the exception of the results of the LIPID trial, the data presented above emerged from relatively small clinical studies, and therefore, results should be interpreted with caution. Moreover, in some cases, more parameters had to be added to the validated scores to improve prognostic value. No data regarding the cost efficiency of these strategies is available.

### 3.2. Cardiac Troponins (cTns)

Cardiac troponins (cTns) are acknowledged biomarkers of myocardial injury, used in different clinical settings, including MI. The newer generations of high-sensitivity cardiac troponins (hs-cTn) assays can detect myocardial injury earlier, allowing a faster and more efficient rule-in or rule-out of patients suspected of non-STEMI ACS [23]. However, the use of troponin levels as a prognostic marker is not currently mainstream in clinical practice.

#### 3.2.1. High-Sensitivity Cardiac Troponin I (hs-cTnI)

Beyond its use for the diagnosis of non-STEMI, hs-cTnI may also have prognostic value, in apparently healthy patients, or in patients who already experienced an ACS. Even in people without known coronary syndromes, small troponin release may be triggered by increased stress on the myocytes, which, in turn, could be related to an increased risk for cardiovascular events. This relationship between hs-cTnI and MACEs, as well as HF, has already been documented in the Framingham Heart Study a decade ago. However, results from the Framingham cohort suggested that cTnI levels did not predict CAD [24].

More recently, similar results were reported from the Busselton Health study. In this cohort-based study, 3939 people, with and without CVD, were followed for 20 years, and increased troponin I levels were associated with a higher risk for fatal and nonfatal CVD events, as well as HF, but were not predictors of CAD in the CVD-free cohort [25].

However, higher levels of hs-cTnI were shown to predict worse outcomes in patients who presented to the emergency department with the suspicion of ACS. In a cohort of 4748 patients, increased troponin predicted a higher number of subsequent hospitalizations for HF over the 156 days of follow-up. The doubling of hs-cTnI levels translated into a 2.80-fold increase in the risk of HF hospitalization [26].

A biomarker substudy of the Thrombin Receptor Antagonist in Secondary Prevention of Atherothrombotic Ischemic Events—Thrombolysis in Myocardial Infarction 50 (TRA 2°P-TIMI 50) trial involving 15,833 patients with stable atherothrombotic disease reported a gradual association between elevated baseline concentrations of hs-cTnI and BNP and risk of HF hospitalization [27]. Moreover, if hs-cTnI and BNP were both elevated (hs-cTnI ≥5 ng/L and BNP ≥100 pg/mL), the risk of hospitalization was higher than for either parameter alone. The addition of hs-cTnI to a clinical risk factor model (including age ≥75, prior HF, type 2 diabetes mellitus, number of vascular beds with atherosclerotic disease, body-mass index, anemia, chronic kidney disease, and hypertension) contributed very little to the predictive performance (C-index 0.90 vs. 0.88, *p* < 0.001) [27].

By contrast, data from the CHOPIN study suggested that the levels of cTnI measured in patients presenting with chest pain in the emergency department did not predict neither mortality, nor MACEs (defined as ED visit or hospitalization for AMI, unstable angina, HF, reinfarction, stroke) during the 180 days of follow-up. Other biomarkers performed better in predicting those outcomes, as described elsewhere in this article [8].

#### 3.2.2. Determinants of High-Sensitivity Cardiac Troponin I

A detailed exploration of the determining factors that contribute to elevated HF-related biomarkers is essential to broaden the understanding of the clinical utility of these assays. A post-hoc analysis of the Scottish Computed Tomography of the Heart (SCOTHEART) trial, including 885 patients with stable chest pain, evaluates the determinants of elevated hs-cTnI and BNP levels [2]. This study reports significant associations between hs-cTnI and increasing age (*p* < 0.001), male sex (*p* = 0.002), indexed left ventricular (LV) mass (*p* < 0.001), and atherosclerotic plaque burden (*p* = 0.007).

#### 3.2.3. High-Sensitivity Cardiac Troponin T

High-sensitive troponin T (hs-cTnT) was investigated in a multicenter observational study, in order to assess its ability to identify patients with lower risk of rehospitalization and mortality at 90 days among those who present to the emergency department with signs or symptoms of acute HF [28]. Based on the findings of this study, hs-cTnT below the 99th percentile was not associated with a significantly lower risk for the composite endpoint and therefore should not be used to justify ED discharge decisions in patients with suspected acute HF.

#### 3.2.4. Troponins as Biomarkers for Incident or Subclinical HF

A number of studies suggest the potential use of biomarkers such as troponin T or NT-proBNP alongside clinical factors in identifying patients that could benefit from preventive therapy before incident HF. A substudy of the Multi-Ethnic Study of Atherosclerosis including 6781 participants with no cardiovascular disease at baseline shows that detectable troponin T was a significant predictive biomarker for new onset HFpEF, consistent across different racial or ethnic groups [5]. Similarly, the Jackson Heart Study investigated 3987 African-American participants free of cardiovascular disease at inclusion and showed that the risk of developing HF later in life was related to myocardial injury as assessed by troponin levels [29]. The study found a dose-dependent association between hs-cTnI at baseline and risk of HF. Moreover, the combination of elevated hs-cTnI and left ventricular hypertrophy predicted the highest risk for progression to HF [29].

Serial measurements of cardiac troponin may also improve the early identification of patients prone to HF. In a prospective cohort observational study, including 8838 patients without HF or CHD, hs-cTnT was measured at enrollment and after six years. Patients were further followed for 14 years [30]. If hs-cTnT levels were <0.005 ng/mL at baseline, and >0.005 ng/mL after six years, the risk of HF was two times higher, while the risk of CHD and death also increased. These results also suggest the potential of hs-cTnT concentration changes in detecting subclinical cardiomyocyte damage, with prognostic implications in initially asymptomatic patients [30].

Moreover, a subgroup analysis of data from the Atherosclerosis Risk in Communities Study (ARIC) (n = 1054) showed that higher hs-cTnT measured years before an MI (six years, on average) is associated with an increased risk of adverse cardiovascular outcomes. The findings of this study show a gradual association of increasingly higher levels of hs-cTnT with a higher risk of incident MI, as well as an association of hs-cTnT with heart failure and cardiovascular death [6]. A different ARIC substudy [31], including 8121 participants free of cardiovascular disease at baseline and followed for 15 years, reported the association of elevated hs-cTnI levels (≥3.8 ng/L) with incident coronary heart disease, hospitalization for HF and global CVD using a risk factor adjusted model. The investigators found only a moderate correlation between hs-cTnI and hs-cTnT levels, which pointed to their complementary roles in risk prediction. Compounded prediction models including both hs-cTnI and hs-cTnT, in addition to the standard pooled cohort equation model (a 10-year risk assessment equation for atherosclerotic CVD), improved the prediction of incident events reflected in increases of AUC compared to the pooled cohort equation model alone (ΔAUC 0.019 for ASCVD, 0.026 for global CVD and 0.035 for HF) [31].

#### 3.2.5. Post-Hospitalization or Post-Procedural Troponin Elevations

The available data regarding risk stratification in patients who are discharged after hospitalization for HF is scarce, as the majority of existing risk scores focus on inpatients. An analysis of findings from the prospective multicenter Aliskiren Trial on Acute Heart Failure Outcomes (ASTRONAUT) randomized trial (including adult patients, hospitalized for worsening CHF, LVEF ≤40%) showed that increased levels of troponin I one month post-discharge were independently associated with a higher rate of all-cause mortality and cardiovascular mortality or HF hospitalization at 12 months [32].

Moreover, Cottens D. et al. attempted to analyze the predictive value of hs-troponin for MACEs in 409 patients with stable CAD who underwent PCI for chronic total occlusion [33]. MACEs were defined as a combined endpoint including mortality, MI, target vessel failure, clinically driven target vessel revascularization and stroke. In this study, 85% of the patients had increased troponin levels, among whom 47% had significant elevations (defined as a 20% postprocedural rise when baseline values were elevated or more than five times the upper limit of normal when baseline results were either in the reference range or unavailable) [33]. Despite the frequency of increased troponin levels, there were no correlations between hs-cTnT elevations and either higher mortality or MACEs after one year post-PCI [33]. The most relevant trials and clinical outcomes are summarized in Table 2.

#### 3.2.6. Point-of-Care Troponin Assays

Pre-hospital determination of cardiac troponin T using point-of-care devices (POC-cTnT) has been proposed for improving the early diagnosis of AMI and identification of high-risk patients. In a large study, on 16,449 patients, Rasmussen et al. tested the use of pre-hospital cTnT, measured on point of care devices, for diagnosing AMI, and predicting outcomes [34]. In this study, routine pre-hospital POC-cTnT diagnosed AMI with 44.2% sensitivity, 92.8% specificity, 44.9% positive predictive value, and 92.6% negative predictive value. Moreover, POC-cTnT ≥50 ng/L was associated with a two-fold increase in mortality during the 2.35 years of follow-up, regardless of the in-hospital confirmation of AMI (HR = 2.10 [1.90–2.33], *p* < 0.001) [34].

#### 3.2.7. Prediction Scores Including Cardiac Troponins

As for BNPs, several authors attempted to demonstrate a contribution of cardiac troponins to multiple-parameter risk prediction models. An analysis from the EXAMINE trial investigated an extended panel of biomarkers, including hs-cTnI, as predictors of HF after recent ACS in 5380 patients with type 2 diabetes mellitus who were followed for 18 months. [17] Although a doubling of hs-cTnI levels was significantly linked to CV death or HF hospitalization, NTproBNP levels were more strongly correlated to these events. An association was found between the primary outcome (defined as CV death, hospitalization for HF, elevated NTproBNP or initiation of loop diuretics) and hs-cTnI (HR 1.04 [1.00–1.09]), galectin-3 (HR 1.21 [1.03–1.41]) and GDF-15 (HR 1.15 [1.04–1.28]). These findings support a multimarker approach for improving HF prognostication in patients with type 2 diabetes mellitus. Another study that proposes a multimarker risk model for incident HF performed a post-hoc analysis of data from the LIPID study (n = 7101 patients with a history of MI or unstable angina) [15]. The study reports an association between incident HF and concentrations of hs-cTnI >0.018 μg/L, BNP >50 ng/L, cystatin C >0.93 nmol/L, D-dimer >273 nmol/L, hs-CRP >4.8 nmol/L and demonstrates that adding these biomarkers to a clinical prediction model improves risk stratification.

Machine learning algorithms applied for identifying prognostic serum biomarkers have an expanding role in the field of cardiovascular outcome prediction and could aid in developing high-quality, robust prognostic prediction tools. Cardiac troponin-T could be used as a predictor of incident heart failure, as shown by an observational study using machine learning techniques in a cohort of initially asymptomatic patients (n = 6814) over a 12-year follow up period [4].

### 3.3. Parameters of the Lipid Metabolism

The implication of the lipid metabolism in the pathophysiology of atherosclerosis is undeniable. High levels of serum lipids are strongly associated with the development of atherosclerosis. Particularly, low-density lipoprotein cholesterol (LDL-C) and low levels of high-density lipoprotein cholesterol (HDL-C) are established risk factors for CAD and its complications such as ACS. Triglycerides are another major component of serum lipids and were shown to be an independent risk factor for CAD. In this section, we present recent literature data on the use of established and emerging lipid metabolism biomarkers for medium- and long-term prediction of mortality and cardiovascular outcomes after MI.

#### 3.3.1. Cholesterol and Triglycerides

High cholesterol, particularly LDL cholesterol, is significantly associated with the process of atherosclerosis. LDL cholesterol levels are the guideline recommended target for treating dyslipidemia, and for secondary prevention of adverse cardiovascular events in patients who already experienced a myocardial infarction or stroke [35]. Ever lower targets have been established for LDL cholesterol over the years, as new lipid-lowering medication has been developed. However, despite so much research in the field, new and creative ideas regarding LDL cholesterol as prognostic marker are always emerging.

For example, LDL cholesterol levels were shown to be predictors of microvascular injury, as assessed by cardiac magnetic resonance imaging two days after the acute event. The study enrolled 235 STEMI patients who were treated by primary PCI within 24 h from admission [36]. In this study, patients displaying microvascular injury had significantly higher levels of total cholesterol (*p* = 0.01) and LDL-C (*p* = 0.001), whereas HDL-C and triglycerides were not significantly different. The rate of microvascular injury increased with increasing LDL-C tertiles: <2.92 mmol/L (<113 mg/dL)—43% microvascular injury; 2.92–3.88 mmol/L (113–150 mg/dL)—55%; >3.88 mmol/L—67%. Over a 20-month follow-up, an LDL-C cut-off value of 3.88 mmol/L (150 mg/dL) was predictive for a three-fold increase in MACE (defined as composite of all-cause death, myocardial reinfarction, and new congestive HF). Moreover, MACE rates increased with increasing LDL-C concentrations: 0% for LDL-C <1.81 mmol/L (<70 mg/dL), 6% for LDL-C 1.81–3.88 mmol/L (70–150 mg/dL), and 16% for LDL-C >3.88 mmol/L (>150 mg/dL) [36].

Non-HDL-cholesterol has recently gained more attention as a valid parameter in the assessment of patients who have atherosclerosis and atherosclerosis-related events [37]. In a study on 1842 patients with a history of MI from the Chronic Heart Failure Analysis and Registry in the Tohoku District (CHART-2) cohort [38], the reoccurrence of MI increased with increasing non-HDL-C levels, but not LDL-C or triglyceride levels alone. Non-HDL-C was not associated with all-cause mortality in either of the groups (<2.58 mmol/L, 2.58–3.36 mmol/L, and >3.36 mmol/L). Patients with LDL-C ≥2.58 mmol/L under statin treatment had higher incidence of recurrent MI regardless of triglyceride levels by comparison with those with LDL-C <2.58 mmol/L and triglyceride ≤1.68 mmol/L (≤149 mg/dL). Moreover, MI recurrence significantly increased with increasing triglyceride levels in patients with both LDL-C <2.58 mmol/L or above [38].

In addition, on a study on 130 hospitalized STEMI survivors, the visit-to visit LDL-C and HDL-C variability were associated with MACE over a mean follow-up period of 62.4 months (starting from two months post-discharge) [39]. In this study, MACEs were defined as death, myocardial infarction, stroke, unplanned revascularization, and HF admission. Compared with non-MACE patients, those who suffered a MACE had higher LDL-C variability, and the risk of MACE increased with increasing LDL-cholesterol variability [39]. In addition to that, MACE patients had a higher mean follow-up LDL-C (*p* = 0.033) [39].

Triglycerides have long been overlooked as predictors of atherosclerosis and cardiovascular events. More recently, the triglyceride-glucose index, calculated as ln[fasting triglycerides (mg/dL) × fasting blood glucose (mg/dL)]/2, emerged as a novel surrogate of insulin resistance, and a risk factor for CAD. In a recent study, 1179 patients with non-obstructive coronary artery MI were followed for 41.7 months, for the occurrence of MACE, defined as all-cause death, nonfatal MI, revascularization, nonfatal stroke, and hospitalization for unstable angina or HF [40]. In this study, a higher triglyceride-glucose index was correlated to a higher risk of MACEs, and the likelihood of an adverse event increased with higher triglyceride-glucose index tertiles [40]. When analysis was performed on subgroups of patients, stratified according to sex, age, body mass index, type of MI, history of hypertension, diabetes mellitus, dyslipidemia, and LDL-cholesterol levels, the triglyceride-glucose index remained a significant risk factor (*p* < 0.05 for all).

#### 3.3.2. Lipoprotein (a)

Despite conflicting evidence, the current view is that higher Lp(a) levels are associated with an increased risk of atherosclerotic CVD [35]. Data from the Atherosclerosis Risk in Communities cohort (n = 14,154, no HF at baseline) showed that Lp(a) was a long-term predictor of HF hospitalization (median follow-up 23.4 years). However, MI morbidity seems to have weighted significantly in the analysis as Lp(a) was no longer associated with HF hospitalization after exclusion of prevalent and incident MI cases [41]. This finding is in line with current pathophysiological models where Lp(a) is primarily involved in plaque development and progression to CHD, while HF is predominantly caused by CHD and its complications. As such, any attempt at associating Lp(a) and HF should take into consideration pre-existing CHD [41].

To date, the postmorbid predictive value of Lp(a) has not been established. Other studies have also reported on the lack of predictive value of Lp(a), albeit on smaller cohorts and after significantly shorter follow-up times. A substudy of the LIPID trial on patients with a history of ACS 3–36 months before recruitment (n = 7101), reported that Lp(a) was not predictive for incident HF over a five-year follow-up period [15]. Another study on 1711 patients admitted with STEMI and followed-up for one year [42] also reported that Lp(a) was not predictive for MACEs (cardiac death, MI, or stroke). Overall, these studies suggest that, despite its established role as an independent cardiovascular risk factor, Lp(a) may be less relevant for the prognostication of clinical outcomes after the onset of cardiovascular morbidity, namely ACS.

#### 3.3.3. Other Lipid-Related Parameters

Vaspin (visceral adipose tissue-derived serpin) is an anti-inflammatory insulin sensitizer adipokine associated with obesity. A study on 1036 patients with acute MI showed that admission fasting levels of vaspin had predictive value for MACEs (CV death, recurrent MI, or HF hospitalization) over a median follow-up of 574 days: cut-off 0.62 ng/mL, AUC 0.785, *p* < 0.001 [43]. Although high vaspin levels were associated with obesity and diabetes mellitus, low vaspin was found to be an independent predictor of MACEs, as well as individual outcomes such as HF hospitalization, recurrent MI, but not CV death. Moreover, the addition of vaspin to the conventional risk factors model significantly improved integrated discrimination (0.072 [0.045–0.126]) and net reclassification (0.098 [0.053–0.164] [43]. Although its seemingly complex metabolic role is yet to be fully clarified, these results suggest that vaspin may prove to be a useful emerging prognostic biomarker in patients with MI.

Seven(7)-ketocholesterol (7-KC), found in atherosclerotic plaques, is a major product of cholesterol oxidation that is reportedly more atherogenic than cholesterol. A study on 1016 patients from the Expanded Guangdong Coronary Artery Disease cohort aimed to investigate the prognostic value of 7-KC in patients with stable CAD [44]. After a median follow-up of 4.6 years, adjusted analysis revealed that patients in the highest quartile of 7-KC had a higher risk of MI, HF hospitalization, CV death, all-cause death, and composite outcome (first of all individual outcomes). The study also revealed that a 1 SD increase in 7-ketocholesterol level was associated with an increased risk of clinical outcomes: 17% for MI, 38% for CV death, 45% for all-cause death, and 36% for the composite outcome. Compared to reference patients with low 7-KC and low LDL-C (both below median), patients with high 7-KC were at increased risk for the composite outcome even in the absence of elevated LDL-C. However, this association was not observed for patients with high LDL-C in the absence of high 7-KC. Furthermore, patients with high LDL-C and high 7-KC are at increased risk for the composite outcome when compared to those with elevated 7-KC and low LDL-C. Thus, it seems that the prognostic value of high 7-KC is independent of LDL-C and can be added to that of elevated LDL-C. Finally, 7-KC improved the integrated discrimination and category-free net reclassification of existing models based on traditional risk factors alone (0.053 [0.026–0.083] and 0.400 [0.230–0.560], respectively) or in combination with NT-proBNP and hs-CRP (0.015 [0.006–0.032] and 0.140 [0.015–0.300], respectively) [44]. The most relevant trials and clinical outcomes are summarized in Table 3.

### 3.4. Inflammatory Markers

Emerging evidence from numerous reports has highlighted the central involvement of inflammation in the development and progression of atherosclerosis. The efficacy of inflammatory markers as instruments in cardiovascular risk stratification varies according to the specific biomarker evaluated and in particular subpopulations and has therefore been thoroughly investigated in multiple clinical scenarios.

#### 3.4.1. High Sensitivity CRP (hs-CRP)

A large-scale study of 17,464 adult MI survivors with at least 1 hs-CRP measurement in the predefined three-month baseline eligibility period shows the critical inflammatory risk in this category of patients [45]. A considerable proportion (66%) of the included patients had hs-CRP ≥2 mg/L in association with an increased risk of major adverse cardiovascular events (a composite endpoint of nonfatal MI, nonfatal ischemic stroke or cardiovascular death). Interestingly, the association is linear only up to hs-CRP <5 mg/L and hits a plateau after this point, alluding to potential clinically relevant implications linked to this cut-off value [45].

The role of hs-CRP as a predictive biomarker for HFpEF arising as a complication of first AMI is investigated in an observational retrospective study of 405 patients undergoing primary PCI [10]. Patients developing HfpEF had higher hs-CRP levels as well as higher white blood cell and neutrophil counts, higher NT-proBNP, peak CK-MB, and troponin I levels compared to patients without post-MI HF. Moreover, hs-CRP and NT-proBNP were the biomarkers identified as independent predictors of in-hospital HFpEF after first MI [10]. Relevant statistical data for this section are presented in Table 4.

**Table 4:** Predictive value of CRP for clinical outcomes in patients with ischemia-related cardiovascular diseases.

Biomarker	Participants	N	Primary Outcome	Median Follow-Up	Cut-Off Value	HR/OR [95% CI]	Ref.
**hs-CRP**	MI survivors	17,464	MACE:nonfatal MI, nonfatal ischemic stroke or CV death	3.2 years	Baseline ≥2 mg/L	HR 1.28 [1.18–1.38]	[45]
	Post-STEMI patients	204	LVSD at 6 months after hospital discharge	5.6 years	≥19.67 mg/L at 24 h after admission	OR 1.47 [1.10–1.97]	[46]
**CRP**	Previous MI patients	2184	All-cause mortality	6.4 years	Baseline ≥2 mg/L and ≥2 mg/L at 1 year	HR 2.12 [1.60–2.80]	[47]
			CV mortality			HR 2.31 [1.48–3.61]	

Abbreviations: CRP C reactive protein, CV cardiovascular, HFpEF heart failure with preserved ejection fraction, HR hazard ratio, hs-CRP high sensitivity C reactive protein, LVSD left ventricular systolic dysfunction, MACE major adverse cardiovascular events, MI myocardial infarction, OR odds ratio, Ref reference, PCI percutaneous coronary intervention.

#### 3.4.2. Kinetic Evaluation of CRP and hs-CRP

The dynamics of hs-CRP levels may provide prognostic information, beyond the measurement of a single value. A report encompassing 2184 patients with previous MI from the Chronic Heart Failure Registry and Analysis in the Tohoku district-2 (CHART-2) study focused on the importance of persistently elevated CRP levels [47]. Patients with CRP ≥2 mg/L at baseline followed by CRP ≥2 mg/L after one year had significantly increased cardiovascular death rates compared to patients with CRP ≥2 mg/L at baseline and CRP <2 mg/L at one year [47].

Moreover, in a prospective cohort study of 204 post-STEMI patients, the authors investigated the ability of serial hs-CRP measurement for predicting long-term post-MI LV systolic dysfunction [46]. For this purpose, hs-CRP was measured four times: at initial hospital admission, after 24 h, at discharge, and at one month after discharge. Higher hs-CRP levels (≥2 mg/L) at 24 h after admission, at discharge, and at one month after discharge were correlated with LV systolic dysfunction, defined as left ventricular ejection fraction ≤40% on echocardiography at six months. The predictive value was increased if hs-CRP was ≥19.67 mg/L at 24 h after admission. These findings highlight the detrimental effects of ongoing inflammation, as shown by constantly increased CRP or hs-CRP levels at baseline and follow up [46].

## 4. Discussion

In this two-part work, we aimed to review recent and relevant literature regarding the prediction of clinical outcomes, mostly in the setting of ischemic CVD, such as ACS and ischemia-related HF. The literature search was focused on both established and emerging humoral biomarkers. It was not the aim of this review to extensively discuss the pathophysiological mechanisms supporting the predictive role of various biomarkers, and therefore data from the literature were summarized, contextualized, and biomarkers were grouped in sections based on category and clinical context in order to facilitate both integrative and selective study.

In the first part of the current work, we approached the rather more conventional biomarkers used in the assessment of patients with CVD. However, our goal was not to describe the research behind the mainstream use of these biomarkers, but to provide information regarding new discoveries, published in the last seven years. There are many clinical studies which focus on biomarkers, because the search for a flawless biomarker is never ending. Unfortunately, at least for the time being, there is no single biomarker that can be considered perfect.

Although there is a plethora of publications, on quite many biomarkers, a definite conclusion, even regarding the multipurpose use of conventional parameters, remains elusive. With a few exceptions, most data on the use of biomarkers come from rather small studies, and study design varies greatly. Moreover, many emerging biomarkers have been proposed, some of them even more promising than traditional biomarkers. These biomarkers will be approached in the second part of the current work.

## Figures and Tables

**Table 1:** Predictive value of natriuretic peptides for clinical outcomes after acute coronary syndrome.

NP	Trial	Participants	Outcome(s)	Follow-Up	N	Predictor/ Cut-Off Value	Risk [95% CI]	Ref.
**BNP**	LIPID	Stable CHD (ACS history)	HHF or HF death	5 years	1729	Increased >50.29 pg/mL	HR 2.04 [1.70–2.46]	[15]
	Hellenic HF Study	ACS diagnosis	Fatal CV event	10 years	1000	Two-fold increase	OR 1.34 [1.10–1.63]	[20]
	EPHESUS	MI + LVSD or DM + MI + LVSD	All-cause death (BNP baseline and 1-month change)	12.6 months	463	Tertile 3 vs. tertile 1 (baseline: >128 vs. <199 pg/mL; 1-month change: >11 vs. −93 pg/mL)	OR 10.90 [2.40–49.5] OR 1.72 [0.81–3.69]	[14]
			CV death or HHF (BNP baseline and 1-month change)				OR 10.10 [3.41–30.2] OR 2.28 [1.21–4.28]	
	ELIXA	DM with recent ACS	HF	26 months	5525	>500 pg/mL	HR 3.00 [2.10–4.10]	[18]
**NT-proBNP**						>700 pg/mL	HR 2.50 [1.70–3.50]	
**(log2)NT-proBNP**	EXAMINE	DM with recent ACS	Composite HF outcome: CV death, HHF, initiation of loop diuretics, or elevated NT-proBNP at follow-up	18 months	5154	Higher	HR 1.24 [1.18–1.31]	[17]
			CV death or HHF			Two-fold increase	HR 1.45 [1.34–1.57]	
**(ln)NT-proBNP**		CAD with recent ACS	All-cause death	52 months	642	Higher	HR 1.53 [1.13–2.07]	[16]
			Cardiac-related death			Higher	HR 1.62 [1.11–2.36]	
			Lower survival			≥290.4 pg/mL	HR 3.79 [1.57–9.15]	
**5-year ΔNT-proBNP**	Heart and Soul Study	Stable CAD	Composite: HF or CV death	4 years (total: 9)	635	Quartile 4 vs. quartile 1 (>222 vs. ≤5 pg/mL)	HR 3.90 [1.10–13.40]	[19]

Abbreviations: ACS acute coronary syndrome, BNP B-type (brain) natriuretic peptide, CAD coronary artery disease, CHD coronary heart disease, CI confidence interval, CV cardiovascular, DM diabetes mellitus, HF heart failure, HHF heart failure hospitalization, HR hazard ratio, LVSD left ventricular systolic dysfunction, MI myocardial infarction, N number of participants in the study, NP natriuretic peptide, NT-proBNP N-terminal prohormone of brain natriuretic peptide, OR odds ratio, Ref reference.

**Table 2:** Predictive value of troponins for clinical outcomes in patients with ischemia-related cardiovascular diseases.

Tn	Trial	Participants	N	Outcome (s)	Median Follow-Up	Cut-Off Value	HR [95% CI]	Ref.
**hs-cTnI**	Busselton Health Study cohort	Population-based cohort	3939	CVD mortality	20 years	Per doubling of baseline level	1.33 [1.23–1.44]	[25]
				Total mortality			1.16 [1.09–1.24]	
				CVD events			1.18 [1.11–1.25]	
				HF			1.44 [1.31–1.58]	
		Suspected ACS	4748	HHF	156 days	Per doubling of baseline level when <URL (women 16 ng/L, men 34 ng/L)	2.80 [1.81–4.31]	[26]
	Jackson Heart Study	No baseline CVD	3987	Incident HF	9.8 years	4–10 ng/L (women), 6–12 ng/L (men)	1.92 [1.41–1.60]	[29]
						>10 ng/L (women), >12 ng/L (men)	3.52 [2.54–4.87]	
	ARIC Study	No baseline CVD	8121	Incident CHD	15 years	≥3.8 ng/L	2.20 [1.64–2.95]	[31]
				HHF			4.20 [3.28–5.37]	
				Global CVD			3.01 [2.50–3.63]	
				All-cause mortality			1.83 [1.56–2.14]	
**hs-cTnT**		No baseline HF or CHD	8838	HF	14 years	baseline <0.005 ng/mL and follow-up >0.005 ng/mL	2 [1.60–2.40]	[30]
				CHD			1.4 [1.20–1.60]	
				Mortality			1.5 [1.30–1.70]	
		Incident MI	1054	All-cause mortality, CV mortality, recurrent MI, HF, or stroke	3 years	≥14 ng/L	1.53 [1.20–1.94]	[6]
						9–13 ng/L	1.27 [1.00–1.61]	
**TnI**	ASTRO-NAUT	Stable patients hospitalized for HF	1469	All-cause mortality	12 months	>0.04 ng/mL at 1 month follow-up	1.59 [1.18–2.13]	[32]
				CV mortality or HHF			1.28 [1.03–1.58]	
**TnT**	MESA	No baseline CVD	6781	New onset HFpEF	11.2 years	>0.01 ng/mL	4.5 [1.90–10.90]	[5]

Abbreviations: AMI acute myocardial infarction, ARIC Atherosclerosis Risk in Communities, ASTRONAUT Aliskiren Trial on Acute Heart Failure Outcomes, BNP B-type natriuretic peptide, CHD coronary heart disease, CHOPIN Copeptin helps in the early detection of patients with acute myocardial infarction, CI confidence interval, CV cardiovascular, CVD cardiovascular disease, ED emergeny department, HF heart failure, HFpEF heart failure with preserved ejection fraction, HHF heart failure hospitalization, HR hazard ratio, hs-cTnI high-sensitivity cardiac troponin I, MACE major adverse cardiovascular event, MESA Multi-Ethnic Study of Atherosclerosis, MI myocardial infarction, OR odds ratio, Ref reference, Tn troponin, TnT troponin T, TRA 2°P-TIMI 50 Thrombin Receptor Antagonist in Secondary Prevention of Atherothrombotic Ischemic Events Thrombolysis in Myocardial Infarction 50, URL upper reference limit.

**Table 3:** Predictive value of serum lipids/lipid-related parameters for clinical outcomes in patients with ischemia-related cardiovascular diseases.

Biomarker	Details	Outcome(s)	Follow-Up	N	Predictor/Cut-Off Value	Risk [95% CI]	Ref.
**LDL-C**	Stable CHD (ACS history)	HHF or HF death	5 years	6909	>2.5 mmol/L (>97 mg/dL)	HR 2.26 [1.07–4.76]	[15]
	First STEMI + PCI	MVI	48 h	235	Higher	OR 1.02 [1.01–1.02]	[36]
		MACE: all-cause death, MI, or CHF	20 months	222	>3.88 mmol/L (>150 mg/dL)	HR 3.09 [1.22–7.87]	
**LDL-C variability**	STEMI survivors	MACE: all-cause death, non-fatal MI/stroke, HHF, or unplanned revascularization	62.4 months	130	Per 0.01 cVIM increase	HR 1.03 [1.00–1.06]	[39]
**HDL-C variability**						HR 1.06 [1.00–1.13]	
**Non-HDL-C**	Chronic HF with a history of MI; treated with statins	Recurrent MI	8.6 years	1842	≥3.36 mmol/L (≥130 mg/dL) vs. <2.58 mmol/L (<100 mg/dL)	HR 2.70 [1.17–6.24]	[38]
**Lp(a)**	ARIC Study (Community study)	HHF	23.4 years	14,154	Quintiles 4 (11.43–22.96 mg/dL) and 5 (23.10–108.23 mg/dL) vs. quintile 1 (0.02–2.41 mg/dL)	HR 1.16 [1.02–1.34] and HR 1.21 [1.06–1.38]	[41]
	Stable CHD (ACS history)	HHF or HF death	5 years	7101	Not predictive	HR not published (*p* = 0.34)	[15]
	ACS + CAG	MACE: cardiac death, MI, or stroke	1 year	1711	Not predictive (≥30 mg/dL, tertile 3 vs. tertile 1, and per 1 SD increase)	HR 1.05 [0.64–1.73], HR 0.82 [0.52–1.28], and HR 0.98 [0.82–1.19]	[42] *
**TyG index**	MINOCA	MACE: all-cause death, stroke, reinfarction, revascularization, UA, or HHF	41.7 months	1179	Higher; tertiles 2 and 3 vs. tertile 1	HR 1.33 [1.04–1.69]; HR 1.64 [1.06–2.53] and HR 1.85 [1.17–2.93]	[40]
**7-KC**	Stable CAD (± previous MI)	MI	4.6 years	1016	Quartile 4 vs. quartile 1 (>109.8 vs. <55.8 nmol/L)	HR 1.62 [1.35–1.98]	[44]
		HHF				HR 1.43 [1.22–1.76]	
		CV death				HR 1.79 [1.46–2.18]	
		All-cause death				HR 1.91 [1.58–2.39]	
		MI, HHF, or death				HR 1.76 [1.42–2.21]	
**(log)vaspin**	STEMI and NSTEMI patients	MACE: CV death, recurrent MI, or HHF		1036	Lower	HR 0.74 [0.48–0.96]	[43]
		HHF				HR 0.58 [0.37–0.89]	
		Recurrent MI				HR 0.72 [0.53–0.95]	

* Retrospective studies. Abbreviations: ACS acute coronary syndrome, CAD coronary artery disease, CAG coronary angiography, CHD coronary heart disease, CHF congestive heart failure, CI confidence interval, CV cardiovascular, cVIM corrected variation independent of mean, HDL-C high-density lipoprotein cholesterol, HF heart failure, HHF heart failure hospitalization, HR hazard ratio, LDL-C low-density lipoprotein cholesterol, Lp(a) lipoprotein (a), MACE major adverse cardiovascular event, MI myocardial infarction, MINOCA myocardial infarction with non-obstructive coronary arteries, MVI microvascular injury, N number of participants in the study, NSTEMI Non-STEMI, OR odds ratio, PCI percutaneous coronary intervention, Ref reference, SD standard deviation, STEMI ST-elevation myocardial infarction, TyG triglyceride-glucose index, UA unstable angina, WBC white blood cell count, 7-KC 7-ketocholesterol.

## Data Availability

Not applicable.
